# Retraction note

**DOI:** 10.3402/ejpt.v4i0.21913

**Published:** 2013-07-19

**Authors:** Naser Morina

**Retraction:** Combining biofeedback and Narrative Exposure Therapy for persistent pain and PTSD in refugees: a pilot study.

Naser Morina, Thomas Maier, Richard Bryant, Christine Knaevelsrud, Lutz Wittmann, Michael Rufer, Ulrich Schnyder, Julia Müller

**Retraction to:** European Journal of Psychotraumatology (2012) 3: DOI: 10.3402/ejpt.v3i0.17660.

The following article has been retracted by the authors due to irregularities they had found regarding compliance with study procedures and data management. Data files comprising details of participants that were not de-identified were sent between study sites. At the Zurich study site, research sessions and interpreter costs were partly charged to the insurance companies and the hospital rather than to the appropriate research account. Data quality, data analyses, and clinical conclusions drawn from the results were not affected.

June 30, 2013 On behalf of the authors *Naser Morina*

